# A review: therapeutic potential of adipose-derived stem cells in cutaneous wound healing and regeneration

**DOI:** 10.1186/s13287-018-1044-5

**Published:** 2018-11-08

**Authors:** Peng Li, Xiutian Guo

**Affiliations:** Department of Anorectal Surgery, Shanghai Municipal Hospital of Traditional Chinese Medicine, Shanghai University of Traditional Chinese Medicine, Shanghai, 200071 China

**Keywords:** Stromal vascular fraction, Adipose-derived stem cells, Clinical trials, Cutaneous wound healing, Regeneration

## Abstract

As the most important barrier for the human body, the skin often suffers from acute and chronic injuries, especially refractory wounds, which seriously affect the quality of life of patients. For these refractory wounds that cannot be cured by various surgical methods, stem cell transplantation becomes an effective research direction. As one of the adult stem cells, adipose-derived stem cells play an indispensable role in the repair of skin wounds more than other stem cells because of their advantages such as immune compatibility and freedom from ethical constraints. Here, we actively explore the role of adipose-derived stem cells in the repair of cutaneous wound and conclude that it can significantly promote cutaneous wound healing and regeneration. Based on a large number of animal and clinical trials, we believe that adipose-derived stem cells will have a greater breakthrough in the field of skin wound repair in the future, especially in chronic refractory wounds.

## Background

In the past decade, adipose tissue has gained a lot of appeal in tissue repair and regeneration due to its diversity of ingredients and functions. It mainly consists of two subcomponents: mature adipose cell and stromal vascular fraction (SVF). Stromal vascular component cells are a collection of various cell components of adipose tissue after enzymatic hydrolysis and centrifugation, including mesenchymal stem cells, endothelial progenitor cells, lymphocyte, smooth muscle cells, keratinocytes, macrophages, and preadipocytes [1]. SVF have been well recognized in recent years and have been widely used in various animal and clinical studies [2–4]. Cutaneous wound healing and regeneration are a complex and dynamic process consisting of three stages, inflammation, proliferative, and remodeling phase. It involves the activation of many biological pathways and the interaction of some soluble media [5]. Recently, stem cells have many advantages such as multi-differentiation potential, homing mechanism, and secretion of bioactive factors, which play an important role in wound healing and regenerative medicine, especially in refractory wounds where surgeries do not work completely. Stem cells include embryonic stem cells, induced pluripotent stem cells, and adult stem cells. Although embryonic stem cells (ESCs) and induced pluripotent stem cells (iPSCs) have also been studied, their research and development are limited owing to ethical issues and clinical applications [6, 7]. Adult stem cells are favored for many reasons due to their immune compatibility and freedom from ethical constraints.

Adipose-derived stem cells that originate from mesodermal layers are adult stem cells that not only have similar potential as other stem cells, but also differentiate into cells of the other two germ layers [8, 9]. Based on telomere length and beta-galactosidase activity, adipose stem cells exhibit similar senescence in bone marrow mesenchymal stem cells, but its proliferation ability is stronger than that of bone marrow mesenchymal stem cells (BMSCs) [10]. Studies have shown that adipose-derived stem cells (ASCs) can maintain a normal diploid karyotype for 100 generations of culture and its yield is 40 times higher than that of BMSCs [11, 12]. In a rabbit skin injury model, ASC-treated wounds exhibit better epithelial regeneration and collagen deposition than BMSCs [13]. ASCs can be commonly used in a variety of tissue engineering studies for ease and abundance of extraction and are also commonly used for diabetic-related refractory wound [14–16]. Not only that, studies have shown that with the expansion of ASCs culture, ASCs do not cause the proliferation of allogeneic T lymphocytes as a result of the reduction of cell surface histocompatibility antigen, thereby inhibiting mixed lymphocyte reaction (MLR), suggesting that compared with other stem cells, ASCs have the advantage of immunocompatibility and are more suitable for autotransplantation [17]. This review aims to introduce the biology of skin and wound healing, the application of adipose-derived stem cells in wound healing, and regeneration in terms of their differentiation potential, paracrine potential, combined biomaterials, hypoxic conditions, and clinical trials (Table 1).

**Table 1:** List of studies on the paracrine and differentiation potential of ASCs for wound repair and regeneration

Topics of the study	Cell source	Isolation method	Animal model	Function	References
Full-thickness cutaneous wound	Inguinal fat pads	Excision	Rat	Epithelial differentiation and secret angiogenic growth factors	[19]
Acute radiation skin ulcers	Inguinal fat pads	Liposuction	Rat	Promote angiogenesis and granulation	[20]
Full-thickness excisional ears	Inguinal fat pads	Dissection	Rabbit	Activated fibroblast phenotype, increased macrophage recruitment, and enhanced granulation tissue formation	[21]
Acute vocal fold wound	Inguinal fat pads	Excision	Canine	Secrete ECM components, particularly elastin	[22]
Dorsal skin	Human subcutaneous adipose tissue	Excision	Rat	Elevate expression of FGF1 and VEGF	[31]
Full-thickness dorsal wounds	Human subcutaneous adipose tissue	Cesarean section	Mice	Regulation of ECM molecules and fibroblast differentiation	[32]
Full-thickness wound	Human subcutaneous adipose tissue	Cesarean section	Mice	Promoting the migration, proliferation of fibroblasts	[33]
Dorsum circular wound	Human adipose tissue	Liposuction	Mouse	Endothelial and epithelial differentiation	[36]
Full-thickness excision wound	Human subcutaneous adipose tissue	Liposuction	Mice	Promote angiogenesis, affect epidermal morphogenesis and dermal remodeling	[37]
Full-thickness wound	Inguinal fat pads	Excision	Mice	Enhance angiogenesis and regenerative cytokine expression	[39]
Hindlimb ischemia	Human adipose tissue	Liposuction	Mice	Enhanced cell survival and paracrine effects	[40]
Full-thickness wound	Gonadal adipose tissue	Excision	Rat	Enhance vascularization and reduce scar	[42]
Full-thickness cutaneous wound	Inguinal fat pads	Excision	Diabetic rat	Enhance angiogenesis and cell proliferation, facilitate regeneration of granulation tissue	[10]
Full-thickness skin defect wound	Epididymal adipose tissue	Dissection	Zucker diabetic	Improve the survival rate of ASCs	[12]
Dorsum soft tissue ischemia model	Inguinal fat pads	Excision	Fatty rat Mice	Enhance ASC migration and angiogenesis	[49]
Non-revascularizable critical limb ischemia patients	Subcutaneous abdominal adipose tissue	Liposuction	Human	Improve trans-cutaneous oxygen pressure and wound ulcers	[50]

## Biology of skin and wound healing

Skin is a soft tissue that accounts for about 8% of the body’s weight. It is an organ with self-healing and renewal functions, including the epidermis, dermis, and hypodermis [18]. The epidermis mainly includes the stratum corneum, stratum granulosum, stratum lucidum, stratum spinosum, and stratum basale. It is sensitive to external stimuli owing to its abundant nerve endings. The dermis is rich in fibroblasts and is responsible for maintaining stability of the skin’s structure and elasticity. The hypodermis consists mainly of fat tissue and blood vessels, which can store energy and maintain body temperature.

Destruction of normal skin tissue structure and functional integrity can lead to the formation of wounds. Wound healing is a dynamic and complex process involving the release of cytokines, growth factors, and chemokines. It mainly includes hemostasis, inflammation, and proliferative and maturity phase [19]. Hemostasis occurs immediately after wound injury, followed by vasoconstriction, thrombosis, release of cytokines, and activation of endogenous and exogenous coagulation pathways. The inflammatory phase occurs in the first 3 days of wound injury, when inflammatory cells migrate to the wound and mast cells release vasoactive factors to dilate vessels, jointly opening up the defensive response of the wound. In this stage, neutrophils play a key role in removing necrotic tissue and bacteria, while macrophages also facilitate the release of inflammatory cytokines, including transforming growth factors, platelet-derived growth factors, fibroblast growth factors, and epithelial growth factors. The proliferative stage is 3–21 days after wound injury, mainly including angiogenesis, granulation tissue formation, collagen deposition, and epithelial formation. The main role of this stage is to complete the filling of the wound. The final stage of wound repair is maturity, including collagen cross-linking, remodeling, and wound contraction, during which fibroblasts and the collagen fibers play an important role. Proper wound maturation can promote wound healing, and any cause that prolongs this stage will result in scarring or hypertrophic scars, even chronic refractory wounds [20].

## Effect of differentiation potential of ASCs on wound healing

### ASCs differentiate into keratinocytes

Wound healing is a complex, multi-cell process in which fibroblasts and keratinocytes play a crucial role in the regeneration of wounds, tissue remodeling, and deposition of extracellular matrix [21]. ASCs can not only differentiate into fibroblasts, keratinocytes, and endothelial cells, but also secrete some cytokines that can promote their proliferation and migration. Ebrahimian found that GFP-positive cells appeared in the epidermis and dermis after injection of GFP-transfected ASCs on the dorsal surface of the rat, and it was able to express two epidermal keratinocyte marker proteins, cytokeratins CK5 and CK14, confirming that ASCs differentiate into keratinocyte to enhance wound regeneration [22]. However, Sivan also found that ASCs can be differentiated into keratinocytes by culturing autologous ASCs in an imitation eco-environment containing fibrin complexes [23].

### ASCs differentiate into endothelial cells

One of the most important reasons for prolonged wound repair is the reduction of blood flow to the wound. The regeneration of endothelial cells is especially important in the wound repair process, especially in refractory wounds such as ischemic diseases and diabetes. Planat-Benard first discovered that adipocytes and endothelial cells share a common origin. ASCs not only participate in the formation of vascular-like structures, but also enhance the neovascularization of ischemic tissue, suggesting that adipocytes can be a source of cells for angiogenesis in ischemic diseases. This study indicated the direction of adipose-derived stem cells in the study of ischemic diseases [24]. Similarly, Cao had also found in in vivo and in vitro studies that ASCs can differentiate into endothelial cells and can improve blood perfusion and angiogenesis in ischemic lower limbs [25]. Nie found that ASCs transfected with GFP not only can differentiate into vascular endothelial cells and epithelial cells to enhance the vascular structure and epithelial formation of wounds, but also can secrete angiogenic factors such as hepatocyte growth factor (HGF) and vascular endothelial growth factor (VEGF) to promote wound angiogenesis [26]. After Huang transplanted ASCs in a rat model with acute radiation skin ulcer, it was found that ASCs can differentiate into endothelial cells and ultimately promote wound regeneration and angiogenesis [27].

### ASCs differentiate into fibroblasts

Fibroblasts are crucial to wound repair and can secrete various bioactive factors to promote wound healing. In the full-thickness injury model of rabbit ears, Hong found that ASC transplantation can activate the fibroblast phenotype, increase the recruitment of endothelial cells and macrophages, and promote the formation of granulation tissue, while BM-MSC reaches less than this effect [28]. In a vocal cord wound repair experiment, Hu found that ASCs can differentiate into fibroblast-like cells under the regulation of connective tissue growth factors, and injected ASC-differentiated fibroblast-like cells in the vocal fold wounds of dog can significantly enhance wound healing [29]. Hypertrophic scars are characterized by excessive proliferation and production of fibroblasts and extracellular matrix deposition. ASCs can not only promote the proliferation and differentiation of fibroblasts in injured wounds, but also inhibit the excessive proliferation and migration of hypertrophic scar fibroblasts and reduce the expression of related cytokines [30].

## Effect of paracrine potential of ASCs on wound healing

### Effects of cytokines secreted by ASCs on wound healing

In addition to the multi-lineage differentiation potential of ASCs, it can secrete a variety of cytokines, growth factors, and chemokines to regulate angiogenesis and immune responses through paracrine mechanisms, ultimately promoting the repair of damaged tissues. Many evidence indicate that ASCs mainly secrete bioactive factors through the paracrine mechanism to promote the proliferation and migration of endogenous cells, thereby stimulating angiogenesis, epithelial regeneration, and wound remodeling [31]. The process of wound repair involves the combined effects of various cytokines, such as transforming growth factor (TGF-β), hepatocyte growth factor (HGF), matrix metalloproteinases (MMP), vascular endothelial growth factor (VEGF), platelet-derived growth factors (PDGF), granulocyte-macrophage colony-stimulating factor (GM-CSF), interleukin family, EGF, FGF, and tumor necrosis factor-α (TNF-α) [32]. Zhao also studied the effects of four cytokines commonly found in ASC-CM on wound healing. He concluded that VEGF, bFGF, and PDGF-AA can promote the migration of fibroblasts, while bFGF and EGF can promote the proliferation of fibroblasts. And compared with a single cytokine, ASC-CM is more obvious for the proliferation and migration of fibroblasts [33].

In the process of wound repair, it is very important for ASCs to secrete angiogenesis-related activity factors to promote the recovery of wound blood. It is a known fact that ASCs secrete a variety of angiogenesis-related cytokines such as GM-CSF, PDGF, SDF-1, VEGF, b-FGF, HGF, TGF-α, MMP, IL-6, and IL-8 [31, 34]. VEGF is the most important growth factor in the process of angiogenesis. It can promote the mobilization, recruitment, and migration of endothelial progenitor cells and ultimately accelerate angiogenesis [35]. HGF, secreted by ASCs, also plays an important role in angiogenesis. It was found that when HGF is inhibited, ASCs cannot induce endothelial cell proliferation and migration [36]. Besides, some cytokines such as IL-6 and IL-8 play an important role in epithelial regeneration of the wound during the proliferative phase. Heo found that TNF-α-activated ASCs might produce pro-inflammatory cytokine IL-6 and IL-8 to promote angiogenesis of wounds and regeneration of epithelium and ultimately to accelerate the healing of skin wounds [37]. Recently, Hsu confirmed that ASC self-assembly spheroids could release more cytokines, such as FGF1, VEGF and migration-related genes (CXCR4, MMP1), confirming that the enhanced angiogenesis effect is exerted through the paracrine mechanism [38].

### Effects of exosomes secreted by ASCs on wound healing

As a kind of paracrine product of stem cells, exosomes have the same functions as their stem cells and are rich in proteins, mRNA, miRNA, and other substances. Adipose stem cell-derived exosomes have naturally become a hot topic of research. Wang believes that intravenous injection of adipose stem cell-derived exosomes can increase the ratio of collagen fiber III to collagen fiber I and the ratio of TGFβ3 to TGFβ1 and improve MMP3 expression of skin fibroblasts by activating the EPK/MARK pathway to promote ECM remodeling, ultimately promotes wound healing and reduces scar formation [39]. Fibroblasts play an important role in the repair of skin wounds. Co-culture of adipose stem cell-derived exosomes with fibroblasts revealed that concentration of exosomes was found to be proportional to the proliferation of fibroblasts and the expression of N-cadherin (Cyclin-1, PCNA). Furthermore, the exosomal concentration of 50 μg/ml promotes the expression of collagen III and collagen I, suggesting that exosomes may be able to promote wound repair by optimizing fibroblasts [40].

## Effect of pretreated ASCs on wound healing

### The effect of three-dimensional culture of ASCs on wound healing

Because of the low survival rate of ASC transplants on the wound, the use of some special pretreatments not only provides a good ecological environment for ASC transplantation and survival, but also promotes the proliferation, differentiation, and paracrine abilities of ASCs and secrete more factors and growth factors. Keratin is a controllable extracellular matrix protein that regulates cell migration, adhesion, and proliferation [41]. Wu found that the transplantation of adipose-derived stem cells from swine on human hair keratin coating significantly enhanced adhesion, proliferation, and cell viability and induced the adipogenic and osteogenic differentiation of ASCs [42]. Silk fibroin-chitosan film as an effective cell repair mixture and stem cell carrier can significantly enhance wound healing. Altman implants ASCs in silk fibroin-chitosan film and transplants the complex on 6-mm-thick skin wounds of the rat. After comparing the treatment effects of the complex, ASC, and the film alone, it was found that the complex group can significantly enhance the healing of wounds and can also differentiate into endothelial cell and epithelial cell components of injured tissues [43].

In stem cell research, three-dimensional cell culture is a novel technique that can provide stem cells with a cell micro-environment similar to that of the body, which can enhance cell-cell communication and accelerate the wound repair by enhancing the proliferation, differentiation, and paracrine capacity of ASCs. Cell sheet (CS) can also provide a three-dimensional microenvironment for stem cells and can lead to prolonged ASC engraftment. Cerqueira transplanted ASC on thermoresponsive and standard cell culture surfaces to construct a stable 3D cell membrane structure and then transplanted this ASC-containing CS structure to the rat skin full-thickness lesion. The results showed that ASC-CS can promote angiogenesis in new tissues and affect epidermal morphogenesis and dermal remodeling [44]. Five percent of Collagen-Amylopectin hydrogel mimics the 3D human dermal collagen microenvironment and is biocompatible with many cells [45]. Garg transplanted ASCs on this hydrogel by using capillary force. It was found that pullulan-collagen hydrogel scaffold not only improves the stemness of ASCs, but also continuously secretes ASCs to the wound and accelerates wound healing and blood vessel regeneration by accelerating the expression of angiogenesis-related genes [46]. Hsu cultured ASCs on chitosan hyaluronic acid membrane to form ASC spheroids and transplanted them on the 15 × 15-mm full-thickness wound of rat’s back. When compared with ASC dispersed single cells which cultured on polystyrene membranes, ASC spheroids were found to express more cytokines (FGF1, VEGF, CCL2) and migration-related genes (CXCR4, MMP1), suggesting that ASC spheroids have a higher angiogenesis rate and faster wound healing. The fluorescent tracer analysis showed that the ASC spheroids were closer to the microvessels, indicating that the ASC spheroids enhance the wound angiogenesis through paracrine effects [38]. Bhang found that compared with monolayer cell culture, ASC spheroid culture can not only promote the secretion of SDF-1α and HIF-1α, but also enhance the secretion of angiogenesis and anti-apoptosis factors, such as HGF, VEGF, and FGF-2 [47]. Previous studies have shown that PHBV (poly (hydroxybutyrate-co-hydroxyvalerate)) structure has a high water-holding capacity and is easily degraded by enzymes and can maintain the moist state of wounds to accelerate tissue regeneration [48]. Zonari cultured ASCs on the PHBV structure and transplanted it into the mouse’s dorsal full-thickness injury model. Compared with the control group, the PHBV group showed higher expression of VEGF, bFGF, and blood vessel density. It is suggestive of neovascularization and can reduce wound scars by reducing TGF-β1 and α-SMA and upregulating TGF-β3 expression [49].

### Effect of ASC combined skin implantation on diabetic wounds

Diabetes mellitus is a major factor leading to chronic refractory wounds. It is not yet possible to achieve the desired results only by artificial skin grafting and adipose stem cell therapy. It is also necessary to use some biological materials and techniques to promote wound repair. Kaisang used Pluronic F127 hydrogel to create a 3D cell microenvironment for ASCs and transplanted ASCs cultured on hydrogel in a model of skin full-thickness injury induced by streptozotocin in diabetic mice, and found that Pluronic F127 hydrogel can promote the colonization rate of ASCs on the wound surface and enhance angiogenesis and cell proliferation, thereby accelerating granulation tissue proliferation and wound healing [14]. The high extracellular glucose concentration in diabetic wounds leads to the accumulation of advanced glycosylation products AGEs. The production of AGEs can induce the apoptosis of ASCs and inhibit the differentiation of ASCs into endothelial cells. This will eventually lead to delayed wound healing [15]. Kato found that the use of cell sheet (CS) could enhance the ASCs transplant rate. He cultured ASCs derived from epididymal adipose fat on a temperature-responsive culture dishes, and then the ASC sheets were transplanted into the posterior cranial wound of Zucker diabetic fatty (ZDF) rats. Covering with artificial skin to prevent drying, it was found that the ASC sheets group can significantly promote wound healing, suggesting that ASC sheets combined with artificial skin can significantly promote wound repair and improve the survival rate of ASCs [16].

## Effect of ASCs on wound healing under hypoxic conditions

It is well known that oxygen content plays an important role in the maintenance of stem cell properties and functions. Many studies have demonstrated that hypoxia (2%) can increase the proliferation and survival of human adipose stem cells compared to normoxia [50–52]. Rehman found that under hypoxic conditions (1%), ASC secretes five times more VEGF than normoxic conditions (21%). This can significantly increase the proliferation of endothelial cells and reduce the apoptosis [31]. Moreover, Hsiao found that the culture of ASC under hypoxia conditions (< 0.1%) significantly increased the levels of VEGF-A and angiopoietin (ANG) compared to normoxia (20%) and confirmed that the effect of ASC-CM on wound angiogenesis is based on the paracrine mechanism [53]. Similarly, Stubbs pretreated ASC with hypoxia (< 0.1%) for 24 h, which significantly increased the expression of hypoxia-inducible factor-1α (HIF-1α) and its downstream gene VEGF-A compared with the control group (normoxia). This ultimately contributed to wound angiogenesis [54]. Under appropriate hypoxia conditions, ASC-CM not only promotes the synthesis of collagen fibers and the migration of fibroblasts, but also upregulates the expression of VEGF and b-FGF. All of these prove that hypoxia can enhance the repair function of ASC on the wound [55]. Thangarajah confirmed that hypoxia not only promotes the secretion of VEGF by ASCs, but also enhances the expression of SDF-1 receptor CXCR. These contribute to ASC migration and angiogenesis [56]. Although hypoxia conditions do enhance ASC proliferation, survival, and paracrine effects, it is unclear which hypoxic concentration contributes to the ASC characteristics. More experiments are still needed in the future to determine the optimal hypoxic concentration to promote wound healing.

## Clinical efficacy of ASCs

Adipose stem cells as a regenerative drug, many studies have confirmed that it can improve tissue damage and functional defects. The first example of ASC-mediated tissue repair was the repair of pediatric skull defects. In recent years, the research on the characteristics of stem cells has made great progress, making it more effective in more fields. Bura treats severe lower limb ischemia by transplanting ASC (10^8^) cells into the internal and external gastrocnemius and anterior compartment of the ischemic leg. The results showed that trans-cutaneous oxygen pressure and wound ulcers improve significantly, intimating that ASC is feasible and safe for non-revascularizable critical limb ischemia (CLI) [57]. Based on the safety and feasibility of the previous phase 1 and 2 clinical trials, Herreros went on to study the ASC combined with fibrin gel for the treatment of complex anal fistula in a phase III clinical trial. He injected (2–6) × 10^7^ cells into the fistula and found that the 1-year cure rate was 50%, but there was no significant differences in the cure rate with the fibrin gel alone group [58–60]. Compared to animal experiments, there are relatively few clinical trials on ASC treatments. Therefore, more accurate and rigorous trials are needed in the future to analyze the therapeutic effects of ASC on the wound.

## Conclusion

Similar to BMSC, ASC is also an adult stem cell. Although its multi-directional differentiation potential is limited compared with ESC and iPSC, ASC can be easily and massively acquired. Due to its immunocompatibility, it can be used more safely and clinically. ASC can not only differentiate into keratinocytes, fibroblasts, and endothelial cells, but also secrete some growth factors to promote wound healing. Many phase I and II clinical trials have demonstrated that ASC is safe and effective for radiation ulcers, Crohn’s disease, and severe trauma, but there are still many limitations about the role of ASC in wound repair. Until now, the separation and extraction of adipose-derived stem cells has not been a consistent solution, and it cannot clearly clarify the mechanism of ASC-enhanced wound repair. ASC clinical trials are few, only stay in the early stages, and still require a large number of clinical trials to prove the efficacy of ASC in wound repair. Clinical trials on ASC are rare and only in the early stages; more clinical trials will be needed in the future to prove the effectiveness of ASC for wound repair. It is believed that in the future, with the in-depth study of the characteristics of ASCs and their mechanism of action, it can significantly improve refractory wounds which are not effectively treated by current drugs and surgeries. With more and more in-depth studies, it is believed that adipose-derived stem cells can make more breakthroughs in the field of skin wound repair in the future, especially in chronic refractory wounds.

## Abbreviations

ANG: Angiopoietin
ASCs: Adipose-derived stem cells
bFGF: Basic fibroblast growth factor
BMSCs: Bone marrow mesenchymal stem cells
CLI: Critical limb ischemia
CS: Cell sheet
CXCR4: CXC chemokine receptor4
EGF: Epidermal growth factor
ESCs: Embryonic stem cells
FGF: Fibroblast growth factor
GFP: Green fluorescent protein
GM-CSF: Granulocyte-macrophage colony-stimulating factor
HGF: Hepatocyte growth factor
HIF-1α: Hypoxia-inducible factor 1α
IL-6: Interleukin-6
IL-8: Interleukin-8
iPSCs: Induced pluripotent stem cells
MLR: Mixed lymphocyte reaction
MMP: Matrix metalloproteinases
PCNA: Proliferating cell nuclear antigen
PDGF: Platelet-derived growth factors
PHBV: Poly (hydroxybutyrate-co-hydroxyvalerate)
SDF1: Stromal cell-derived factor 1
TGF-β: Transforming growth factor
TNF-α: Tumor necrosis factor-α
VEGF: Vascular endothelial growth factor
ZDF: Zucker diabetic fatty rats
α-SMA: α-smooth muscle actin
